# The telovelar approach reshaped: a new perspective from inside the fourth ventricle

**DOI:** 10.1007/s00381-026-07188-x

**Published:** 2026-03-05

**Authors:** Pierluigi Longatti, Alessandro Fiorindi, Francesca Siddi, Alessandro Boaro, Giuseppe Canova, Alberto Feletti

**Affiliations:** 1 Department of Neuroscience, University of Padova, Padua, Italy; 2 Department of Medical and Surgical Specialties, Radiological Sciences and Public Health, Neurosurgical Unit, University of Brescia, Spedali Civili, Brescia, Italy; 3 Institute of Neurosurgery, Department of Neurosciences, Biomedicine, and Movement Sciences, University of Verona, Verona, Italy; 4 Unit of Neurosurgery, Neuro-Cardio-Vascular Department, Azienda AULSS 2, Marca Trevigiana, Treviso, Italy

**Keywords:** Telovelar approach, Inferior medullary velum, Fourth Ventricle, Posterior fossa, Cerebellomedullary fissure, Anatomy

## Abstract

**Purpose:**

In recent decades, trans-cerebellomedullary fissure (trans-CMF) approaches have gained prominence in fourth ventricle surgery and are largely based on cadaveric anatomical studies of normally sized ventricles. In clinical practice, however, the fourth ventricle is often dilated and distorted by tumor growth. Recent endoscopic in vivo anatomical studies depicting the internal surfaces of both normal and enlarged fourth ventricles have prompted a reassessment of traditional schematic representations of trans-CMF approaches. This refinement does not introduce new surgical strategies but aims to provide neurosurgeons with a realistic in vivo perspective of the ventricular roof prior to its incision across different ventricular sizes.

**Methods:**

Very selected panoramic endoscopic views of the inner roof of twelve cases of both hydrocephalic dilated and normal fourth ventricles were reviewed and analyzed.

**Results:**

The anatomical landmarks were identified, and the schematic cutlines of telovelar procedures were redesigned according to the classical diagrams by Matsushima. In this way, realistic models of roof dissections viewed from inside were obtained, indicating the safest and less safe tracks of the telovelar approach.

**Conclusion:**

This study has enabled the anatomical representation of the internal surface of the roof of very dilated fourth ventricles, creating a more realistic model that better aligns with the clinical realities of fourth ventricles dilated and deformed by tumors. By replicating the classic trans-CMF dissections on these internal roof images, the study provides a more comprehensive and reliable anatomical understanding of the approach.

## Introduction

Trans-cerebellomedullary fissure (trans-CMF) approaches have been developed as a viable technique to safely access and remove tumors and other lesions in the fourth ventricle [1–11]. These advancements ensued comprehensive laboratory studies that focused on dissecting the posterior cerebellar fissure to expose the lower half of the fourth ventricular roof [2, 12–14]. Nonetheless, cadaveric fourth ventricles have some limitations, the main being that they are of normal size and are not comparable to the enlarged, morphologically changed fourth ventricles as they happen during tumour growth. On the other hand, traditional microsurgery does not provide direct visualization of the inner surface of the caudal roof in vivo. This limitation is evident in published schematic images depicting the various opening procedures for trans-CMF approaches. Indeed, the cut lines are typically illustrated on the external surface of the fourth ventricle roof [2, 4]. The absence of a comprehensive, realistic anatomical inside view of the ventricular roof may have contributed to the hesitance some neurosurgeons feel during these procedures. After the tela choroidea is identified and sectioned, cutting the inferior medullary velum (IMV) can raise concerns due to its proximity to the sensitive fastigium zone, and some experts argue that dissection of the IMV may not be necessary, as sufficient access can be achieved through the incised tela alone [4, 11, 15–18] 

**Fig. 1:**
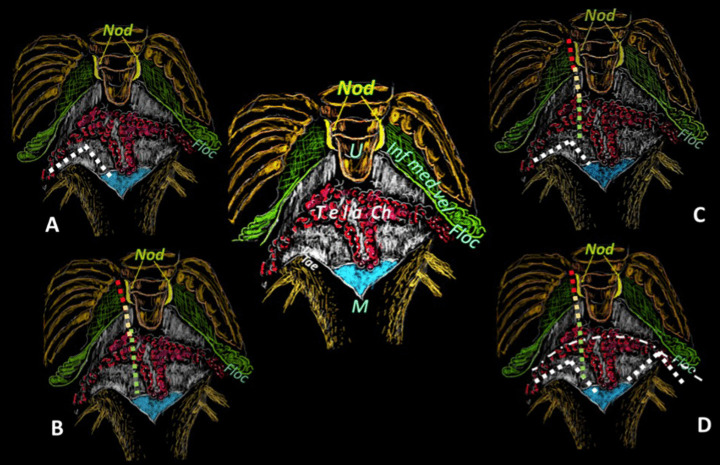
These sketches resume the cerebellomedullary fissure approaches upon the external surface of the fourth ventricle and shall be the map for designing the same dissection lines on the ensuing in vivo endoscopic images of the internal surface of the roof. Central drawing shows the external surface of the caudal roof of the fourth ventricle without the tonsillae and the biventral lobules, the upraised vermian uvula (U) conceals behind the nodulus (Nod in yellow). From that vermian structure, the inferior medullary velum (Inf med vel) branches off bilaterally (area in green), ending with its flocculus (Floc) just outside Luschka’s foramina. Caudally, the tela choroidea (white) with the choroid plexus in red color affixed to the taeniae in white (Tae) close to the Magendie foramen (blue). **A** The resection of the taenia’s longitudinal and horizontal parts opens the Luschka foramen (white dotted lines), allowing the so-called lateral access type. **B** The telovelar approach through the Tela and Plexus (green dotted line), then the tela again (yellow dotted line) toward the IMV (red dotted line). **C** The so-called lateral wall type with telovelar opening. **D** The telovelar and bilateral lateral egress dissection, also called “extensive type opening”

To enhance understanding, it would be beneficial to illustrate these schematic cuts on realistic images of the inner surface of the roof to gain insight into the anatomy from the opposite perspective inside the ventricle. Recent endoscopic studies providing in vivo anatomical descriptions of the inner surface of the fourth ventricle’s roof suggest that these images could be valuable references for illustrating various CMF approaches, particularly the complex telovelar one [17, 18]. That could be accomplished by overlaying the cut lines defined by Matsushima onto endoscopic images of the inner surface of the roof (Fig. 1). The present work aims to enrich the purely anatomical study by providing a more applicable perspective on CMF approaches, potentially boosting neurosurgeons’ confidence during these procedures. Additionally, better insight into the internal anatomy could refine strategies for disconnecting the inferior roof from the brainstem, making tumor debulking within the fourth ventricle easier.

## Methods

**Fig. 2:**
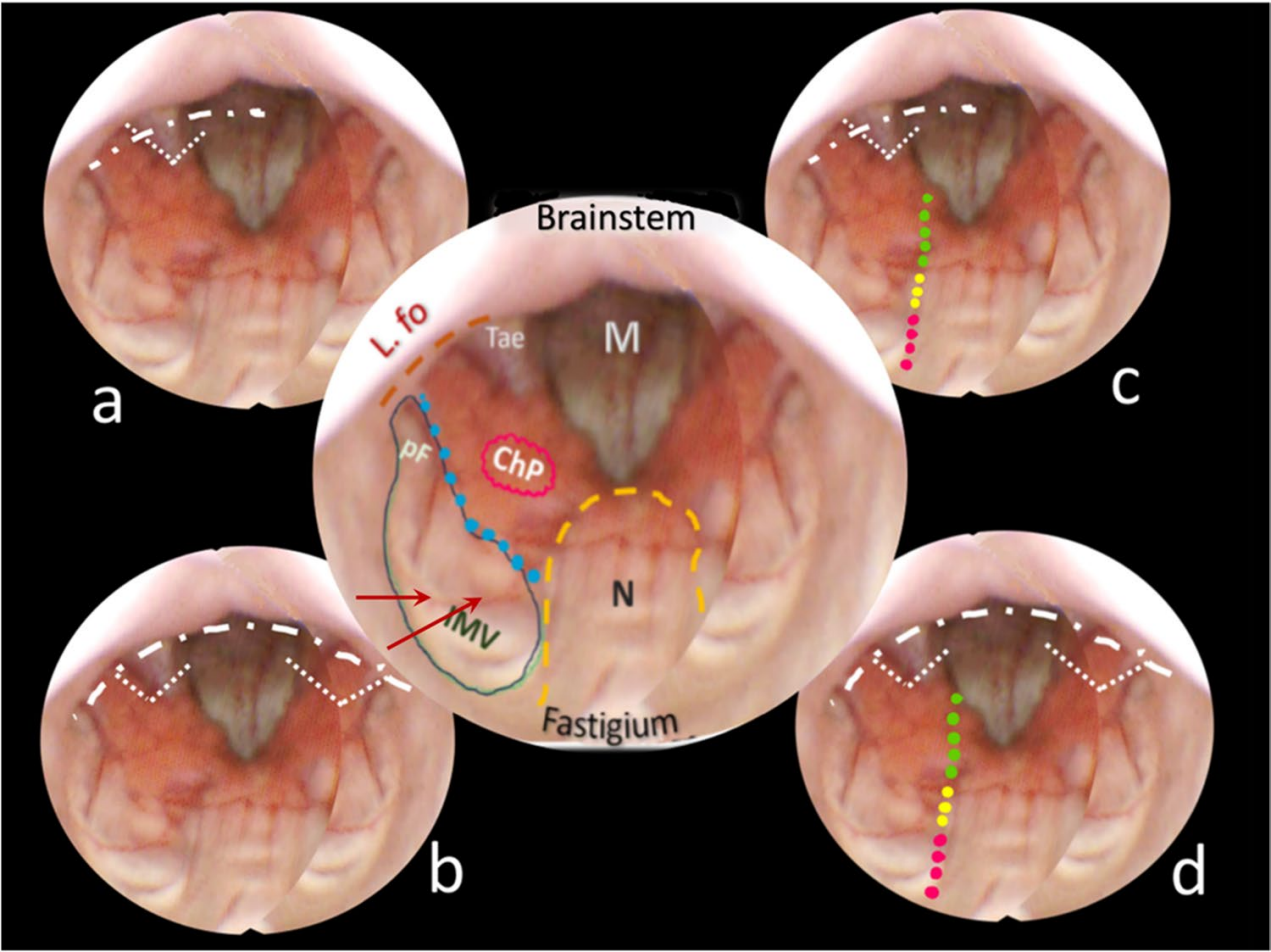
Central round: an anatomical mapping of almost the entire fourth ventricle. The image is captured once the endoscopic camera tip has emerged from the aqueduct and is moving caudally. The nodulus (N, orange dashed line) lies close to the fastigium. Other visible structures are the inferior medullary velum (IMV), outlined in a green loosely dashed line, in part covering the overlying tonsilla, its pedunculus flocculi (pF) directed towards the foramen of Luschka (L.fo), the possible telovelar junctional vein (two small red arrows); the densely dashed-dotted blue line close to the tela and the choroid plexus outlines the telovelar junction. Tela is anchored caudally to the brainstem by the two resilient teniae (Tae) that flank the foramen of Magendie (M) on either side, and cranially, their horizontal segment delineates the L.fo. **A** “Lateral recess type” approach, the dissection of the vertical and horizontal tracts of the taenia (loosely dotted lines) detaches the ventricular roof from the brainstem, connecting the M to the L.fo with a wide opening on the lateral part of the ventricle (densely white dash-dotted line). **B** The effect of bilateral incision of the Tae is more pronounced. **C** “Lateral wall type”: the dissection of the Tae is coupled to the telovelar approach (dotted line). The different colors express the caution advised getting closer to the fastigium. **D** “Extended type” is the combination of different procedures that should open the largest opening to the fourth ventricle

**Fig. 3:**
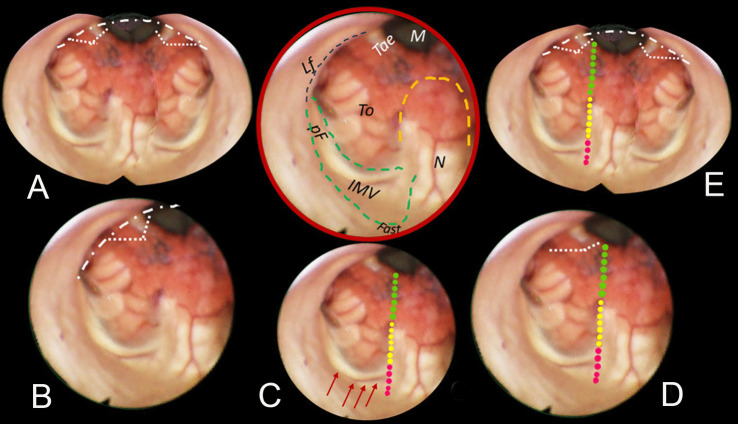
Brown-bordered upper central round: anatomical perspective of almost the entire fourth ventricle. The nodulus (N, orange dashed line) and the inferior medullary velum (IMV, green dashed line) with its pedunculus flocculi (pF) turning into the foramen of Luschka (L.fo) are clearly seen. The tela-velar junction is outlined by the green dotted line separating the IMV and the tonsilla (To). The teniae (Tae) and the foramen of Magendie (M) are also visible. **A** Bilateral complete dissection of both the Taeniae (loosely dotted lines) would create a large opening of the inferior roof (densely white dash-dotted line). **B** In the “Lateral recess type” approach, the dissection of the vertical and horizontal tracts of the Taenia detaches the ventricular roof from the brainstem (white dashed line). **C** The telovelar approach (dotted line). The part of the tela covered by the choroid plexus is less than half the length of the dissection line. The red arrows indicate the telovelar junction vein. **D** In the “Lateral wall type”, the dissection of the Tae is coupled to the telovelar approach (dotted line). The different colors express the caution advised getting closer to the fastigium. **E** The “Extended type” is the combination of different procedures that can provide the largest opening into the fourth ventricle

**Fig. 4:**
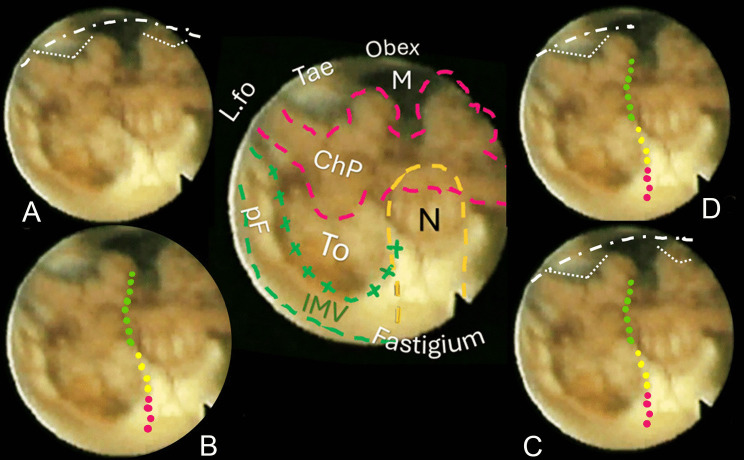
Central sketch: anatomical morphology of the roof of the fourth ventricle. The nodulus (N, orange dashed line) lies caudal to the Fastigium, as well as the inferior medullary velum (IMV, outlined in green loosely dashed line), with the pedunculus flocculi (pF) towards the foramen of Luschka (L.fo). The transparent sleeve over the tonsil (To) can be considered the cranial part of a stretched tela, and the green crossed lines outline the tela-velar junction. Caudally, the tela is overshadowed by the choroid plexus (Chp) and is anchored to the brainstem by the two resilient teniae (Tae) that flank the foramen of Magendie (M) on either side. Cranially, their horizontal segment delineates the L.fo. **A** “Lateral recess” approach: section of the vertical and horizontal tract of the taeniae (white loosely dotted lines) that allows detaching the roof from the brainstem, connecting the Magendie to the Luschka foramina with a wide opening on the lateral part of the ventricle (densely dashed-dotted line). **B** The effect of bilateral incision of the teniae is more pronounced. **C** “Extended type” approach. **D** “Lateral wall type” approach

**Fig. 5:**
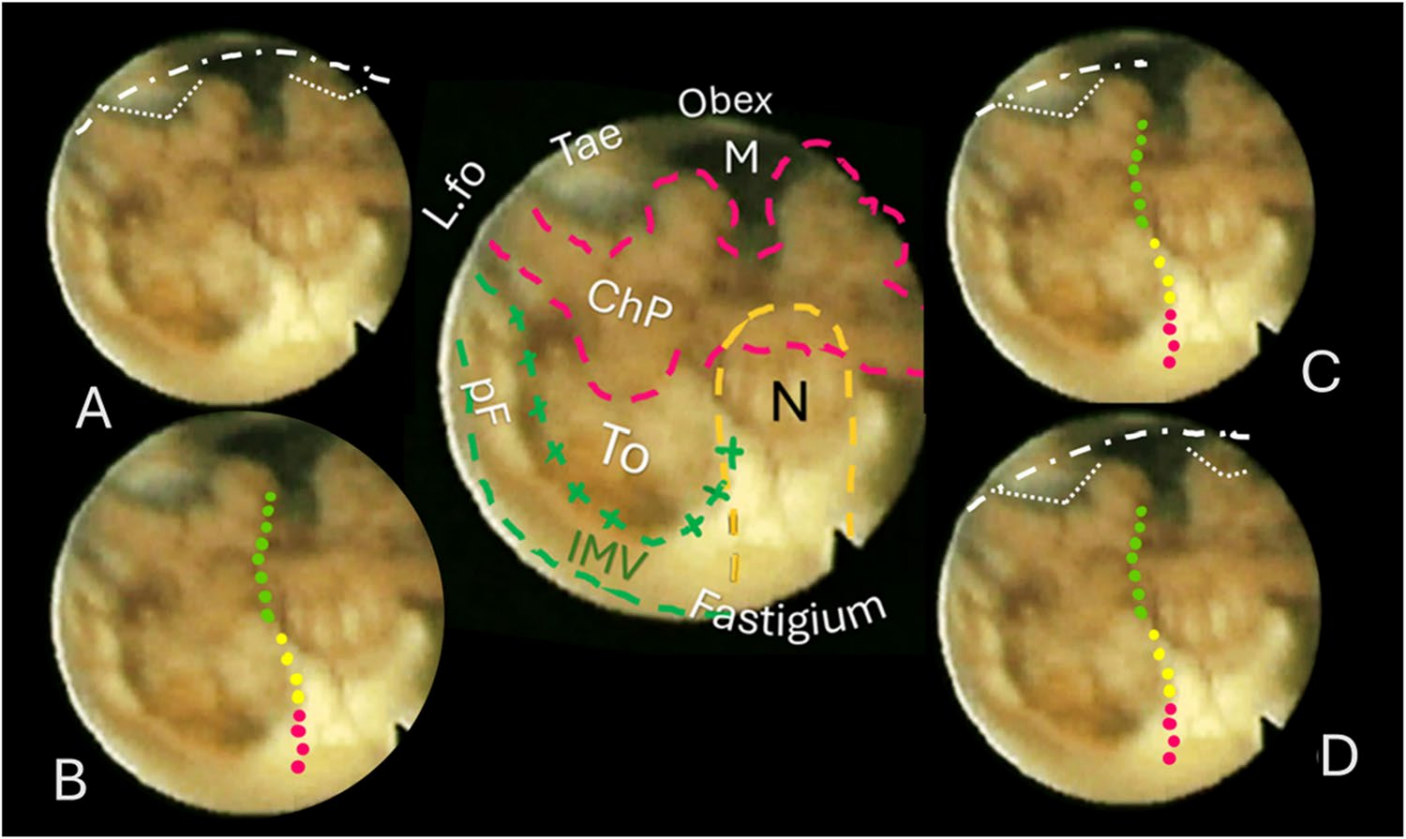
The case of a normal pressure hydrocephalus. Ventricular distention caused an apparent downshift of the choroidal plexus and distension of the cranial part of the roof with enlargement of the inferior medullary velum (IMV), where two components, white and translucent, seem evident. **A** Bilateral taeniae dissection allows access to the ventricle up to the caudal nodulus (N). **B** The telovelar approach, with the green safe dotted line covering about half of the telovelar dissection, is sufficient to reach about the border of the fastigium. The rostral apex of the tonsilla (To) also indicates the limit of a safe telovelar dissection. **C** “Lateral wall type” access. **D** “Extended type” access

**Fig. 6:**
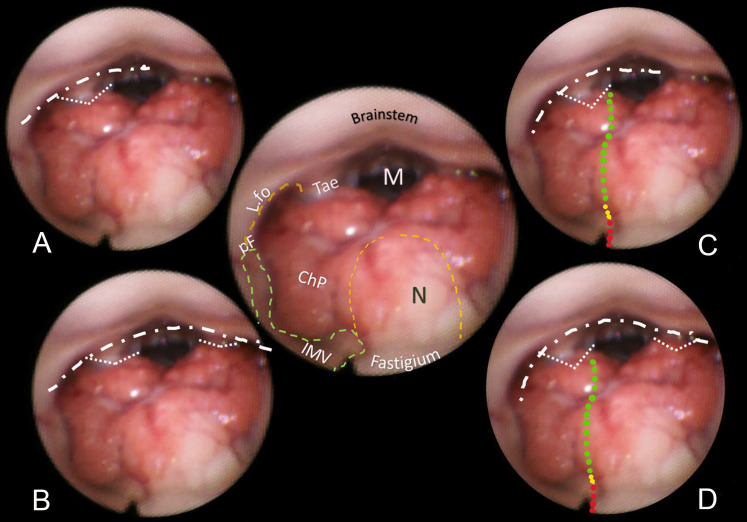
Central round: view of a normal fourth ventricle after endoscopic aqueductoplasty in a 61-year-old man, and comparison with previous figures for anatomical morphology and shortcuts. **A** “Lateral recess” approach: section of the vertical and horizontal tract of the taenia (Tae, loosely dotted lines) that involves detaching the roof from the brainstem (densely dash-dotted line) and connecting the M to the L.fo with a wide opening on the lateral part of the ventricle. **B** The effect of bilateral incision of the teniae is more pronounced. **C** “Lateral wall type” approach. **D** “Extended type” approach: In telovelar procedures, the green-colored dotted line of the tela is prevalent, indicating a safe dissection, while the yellow and red lines are significantly less extended, advising some caution

**Fig. 7:**
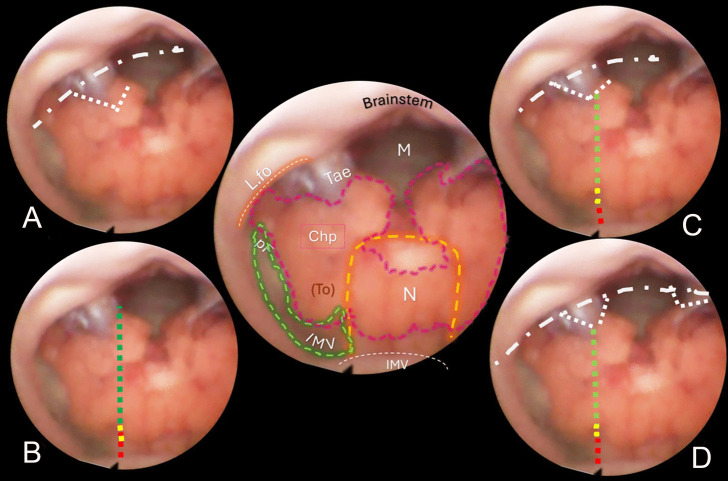
Central round: shape of a normal fourth ventricle after endoscopic aqueductoplasty. The ventricular inferior roof is prevalently dwelt in by the choroid plexus (Chp) that reaches the fastigium. The length of the inferior medullary velum (IMV) appears very short. **A** The “lateral recess” approach: section of the vertical and horizontal tract of the taenia (Tae loosely dotted lines), almost doubling the size of the Magendie foramen opening. **B** The telovelar approach: the green-colored dotted line of the tela indicates a prevalent safe dissection, while the yellow and red lines have a minimal extension, suggesting some caution. **C** The “lateral wall type” approach. **D** The “extended type” approach

Out of our collection of video-recorded procedures of endoscopic transaqueductal navigation of the fourth ventricle, some exemplary paradigmatic cases were identified to resemble either dilated or normal fourth ventricles to illustrate the dissection lines expected in the CMF approaches. Group A includes eight hydrocephalic cases presenting a significant dilatation of the fourth ventricle that could mirror the morphology of the roof when ventricular tumors develop and enlarge it (Figs. 2, 3, 4, 5). Group B encompasses four patients with membranous aqueductal occlusion who underwent endoscopic aqueductoplasty and subsequent exploration of the fourth ventricle. Images were selected as illustrative of normal ventricles (Figs. 6, 7).

The technique for transaqueductal navigation of the fourth ventricle using flexible scopes has been previously documented [18, 19]. In choosing the video recordings, images with acceptable resolution were required, along with the broadest view of the ventricular roof, spanning from the fastigium to the obex and both Luschka foramina. This circumstance is quite rare since the tip of the flexible scope, after entering the fourth ventricle, is often spontaneously turned toward the side of the cavity opposite to the lateral ventricle from which the scope comes [19]. The other side, when explored, is at close range, and therefore, images are deprived of the more important characteristic for this work, which is the panoramic view of the entire ventricular cavity. Eleven morphological items (fastigium, nodulus, inferior medullary velum IMV, pedunculus flocculi, Luschka foramen, tonsilla, telovelar junction, choroidal plexus, taenia, magendie foramen, obex) were identified and delimited in the selected images (Figs. 2, 3, 4, 5, 6). Beyond the morphology of the caudal fourth ventricle roof, we focused our attention on those parts of the CMF that the literature indicates to be dissected: of particular interest were the two taeniae, the tela covering the sulcus between the nodulus and the tonsilla, the telovelar junction, and the IMV. On each image, the incision lines for these approaches were drawn symmetrically to the external schemes proposed by Matsushima (Fig. 1) [2].

## Results

The two groups of normal and dilated ventricles clearly showed different morphologies of the roof. In dilated ventricles, the stretching effects on the fourth ventricle roof were exemplary of what could happen during tumor development and enlargement (Figs. 2, 3, 4, 5, 6). The tela appears more stretched compared to the IMV, and the fastigium looks round-bottomed with a clear definition of anatomical morphology. In normal ventricles (group B), the choroid plexus is dominant in the roof, while little room is left for the IMV (Figs. 6, 7).

The alternative approaches to the classic transvermian incision, as indicated by Matsushima, involve two distinct surgical actions: the resection of the teniae (vertical and horizontal parts) with the opening of the Luschka foramina and the more demolitive telovelar approach [2]. These cutting lines, either considered alone or combined, devised the different approaches depending on the tumor volume, position, and consistency [2, 4, 11]. To reproduce the lateral approach viewed from inside, we traced the lines of incision at the level of the teniae, including the horizontal tract bordering the Luschka foramen. The dissection of both the teniae can enlarge four or five times the size of the Magendie foramen, depending on the normal or dilated fourth ventricles. In any case, the caudal roof of the ventricle should be released substantially. The line reproducing the telovelar approach starts caudally from the resection of the tela once the uvulo-tonsillar fissure has been opened. The marks of the telovelar incision traced from the inside of the roof have been classified into: (1) safe (the most caudal portion colored in green), (2) cautious (from the choroid plexus up to the telovelar junction colored in yellow), and (3) very cautious (the incision of the IMV, colored red) (Figs. 1, 2, 3, 4, 5, 6, 7). The difference between normal and dilated ventricles is significantly interesting: the safe green line transecting the choroid plexus is absolutely prevalent in normal ventricles (group B), while in group A, there is an enlargement of the yellow line, a possible neutral zone between the tela and IMV around the so-called telovelar junction.

## Discussion

Laboratory studies have played a crucial role in advancing trans-cerebellomedullary fissure (trans-CMF) approaches [2–4, 7, 10–12]. However, the present work demonstrates that in vivo endoscopic images of the inner surface of the fourth ventricle roof could be a complementary and advantageous resource, as Matsushima recently commented in his final remarks [4]. In this study, we present cases with normal and dilated fourth ventricles, which can be considered paradigmatic models of the structural ventricular enlargement caused by growing tumors. These distending effects primarily affect the roof, particularly its inferior component, which is composed of various complex membranes on each side of the central vermian nodulus (Figs. 2, 3, 4, 5). Debulking ventricular tumors mostly requires the dissection of this inferior part of the ventricular roof, limited cranially by the impenetrable border of the fastigium, receptacle of the cerebellar nuclei [20].

The cerebellar vermis could be, in theory, an interesting corridor for surgical approaches, being a watershed line of both ascending PICAs and, therefore, relatively vascularized in the middle; consequently, the vermis vertical incision should neither trespass laterally nor should it reasonably intrude superiorly into the fastigium area [11, 16]. Classically, the median transvermian approach has been the accepted procedure to operate on fourth ventricle tumors, and the dissection is strictly limited to the vermis, mainly through the uvula but also deeper through the nodulus, which blends in the fastigium. Vermis and fastigium are structurally indiscernible, and during the operation, this border could inadvertently be trespassed, an event deemed by some authors a source of complications [11, 16, 21].

Alternative to the transvermian opening of the fourth ventricle are the trans-cerebellomedullary fissure (trans-CMF) approaches introduced by Matsushima and Rhoton. They defined the CMF as the posterior roof of the fourth ventricle [4]. There are relatively simpler procedures involving the sectioning of taeniae, resilient ligaments that anchor the tela to the inferior part of the fourth ventricle roof, thereby connecting it to the brainstem. Their dissection should comprise both the longitudinal (8.9 mm) and horizontal (18.4 mm) segments, the latter being the caudal wall of the foramen of Luschka. These incisions do not cause neurological deficits [2, 4, 9, 10, 22, 23].

Opening a single taenia may be enough to remove very lateral lesions (Figs. 2, 3, 4, 5).[2, 21] However, the opening could also be bilateral, thereby granting access to the fourth ventricle, which could become four times larger than the measured size of the Magendie foramen, estimated to be between 4.0 and 9.4 mm (Figs. 2, 3, 5) [2, 9, 24]. On the other hand, this theoretically wide access to the ventricle would only allow a very moderate uplifting of the roof against the brainstem as the PICA crosses the operative field and could become stretched.

Taeniae resections are often combined with the telovelar approaches, involving the opening of the inferior part of the roof of the fourth ventricle starting caudally from the deep uvula-tonsillar fissure, where the tela with the choroidal plexus can be easily identified and safely split [2, 11]. The concluding phase entails the meticulous sectioning of the IMV until the border of the fastigium. As previously noted, some authors discourage this final step, considering the telar dissection sufficient [2].

The endoscopic images presented in this study offer valuable insights. As depicted in Figs. 2, 3, 4, and 5, in cases of dilated fourth ventricles, the stretching appears to predominantly affect the inferior portion of the ventricular roof, particularly the tela choroidea, rather than the IVM. This is further evidenced by the cerebellomedullary fissure vein, which anatomically delineates the boundary between the IVM and the tela, known as the telovelar junction (Figs. 2 and 3). Additionally, the images reveal that the dilation of the tela is not uniform; it primarily involves its cranial segment, which becomes more delicate or transparent over the overlying tonsil. This asymmetric dilation of the tela results in an apparent downward displacement of the choroid plexuses.

Laboratory studies confirmed that once the choroid plexus and tela have been sectioned, the additional opening of the velum provides access to the superior half of the roof of the ventricle until the fastigium, indicating this final velar incision is necessary [5]. Other authors ruled out this absolute need, an opinion confirmed also by our endoscopic images showing that the tiny size of the velum, shaped as a butterfly and measuring in length aside the nodulus barely 6 mm (range 5.5–7.2 mm), does not contribute to a significant widening of the access to the ventricle when it is dissected [4, 25, 26].

During the telovelar approach, the choroid plexus serves as a crucial guide, and its resection, along with the tela, is considered safe [4, 16]. In the surgical practice, a shared experience reveals that when the fourth ventricle is opened to debulk a tumor, once the layer of the tela covering the choroid plexus has been completely dissected, it becomes hard to discriminate the tela from the IMV, or even to precisely determine how deeply the roof has been opened. Only at the end of the surgical debulking, anatomical details such as the proximity to the Fastigium or its violation can be discerned. The high variability in the incidence of postoperative neurological complications reported in series adopting telovelar approaches reflects the significant differences in the operating field, where the fourth ventricle is completely altered in shape and dimensions compared to the laboratory, where the normal anatomy of the fourth ventricle has been studied [7, 16, 21].

In dilated fourth ventricles, the safe zone of the tela with plexuses, denoted in all the figures with a dotted green and yellow line, is sometimes less than 70%. In contrast, the same tracks evaluated in normal ventricles constitute a large part of the pathway up to the fastigium. The problem, when transferred to the operative field, is not so simple. The advancing debulking of the tumoral mass can help the neurosurgeons to understand what tissue they are dealing with, but whenever the plexus has been wholly sectioned, it is advisable to proceed with caution. Other possible anatomical reference points that could help determine when to stop opening the fourth ventricle roof include the tonsillar apical pole, which tapers toward the fastigium (Figs. 2, 3, 4, 5, 6, 7), as well as the cranial loop of the PICA, which was not observed in our images from inside the ventricle.

Endoscopic views of fourth ventricles with normal size, obtained from patients subjected to aqueductoplasty and subsequent inspection of the ventricular cavity, provided a reliable model of a normal, unaltered fourth ventricle, as shown in laboratory specimens, using the same endoscopic technique [27, 28]. Endoscopic panoramic views of the internal surface of the normal fourth ventricle compared with the dilated ones show a different anatomical layout. The choroidal plexus with the nodulus appears to be a cumbersome structure of the inferior roof, and the IMV seems confined in a very narrow fissure. The telovelar line, as traced on the endoscopic images, is largely marked on the plexus and is defined as relatively safe, covering approximately 90% of the length of the telovelar section line. Undoubtedly, the following dissection of the IMV is possible and could be easier than in dilated ventricles, conserving its normal shape, without being stretched; however, it would increase risks without a significant widening of the opening of the fourth ventricle.

In addition to these invaluable data on the mapping of both normal and dilated fourth ventricles, flexible transaqueductal endoscopy could, with the aid of appropriate technology, play a role in assisting microneurosurgery in the future. For instance, this study has demonstrated that the aqueduct serves as a privileged observation point for the fourth ventricle, particularly in areas that are blind and unexplorable by microsurgery, such as the inferior part of the roof. This suggests the potential utility of intraoperative endoscopic monitoring during the debulking of tumors located in the fourth ventricle.

## Conclusions

In conclusion, augmenting the anatomical knowledge by bridging the gap between laboratory classical studies and the faithful, realistic anatomical representation in vivo offered by neuroendoscopy, neurosurgeons may find a more streamlined pathway to successfully navigate the challenges associated with tumors in the fourth ventricle, with a substantial benefit in surgical planning and performance of the CMF approaches.

## Data Availability

No datasets were generated or analysed during the current study.
