# Correction: The telovelar approach reshaped: a new perspective from inside the fourth ventricle

**DOI:** 10.1007/s00381-026-07285-x

**Published:** 2026-05-02

**Authors:** Pierluigi Longatti, Alessandro Fiorindi, Francesca Siddi, Alessandro Boaro, Giuseppe Canova, Alberto Feletti

**Affiliations:** 1 Department of Neuroscience, University of Padova, Padua, Italy; 2 Department of Medical and Surgical Specialties, Radiological Sciences and Public Health, Neurosurgical Unit, University of Brescia, Spedali Civili, Brescia, Italy; 3 Institute of Neurosurgery, Department of Neurosciences, Biomedicine, and Movement Sciences, University of Verona, Verona, Italy; 4 Unit of Neurosurgery, Neuro-Cardio-Vascular Department, Azienda AULSS 2, Marca Trevigiana, Treviso, Italy

**Correction: Child's Nervous System (2026) 42:96**

10.1007/s00381-026-07188-x

In this article, Fig. 2 “Brainstem” was misspelled as “Braistem”. Figure 4 legend, it is written "C) “Lateral wall type” approach. D) “Extended type” approach”; however, it is actually the opposite: “C) “Extended type” approach. D) “Lateral wall type” approach”. In Fig. 5, the letters identifying each part of the figure are missing. This is now added.
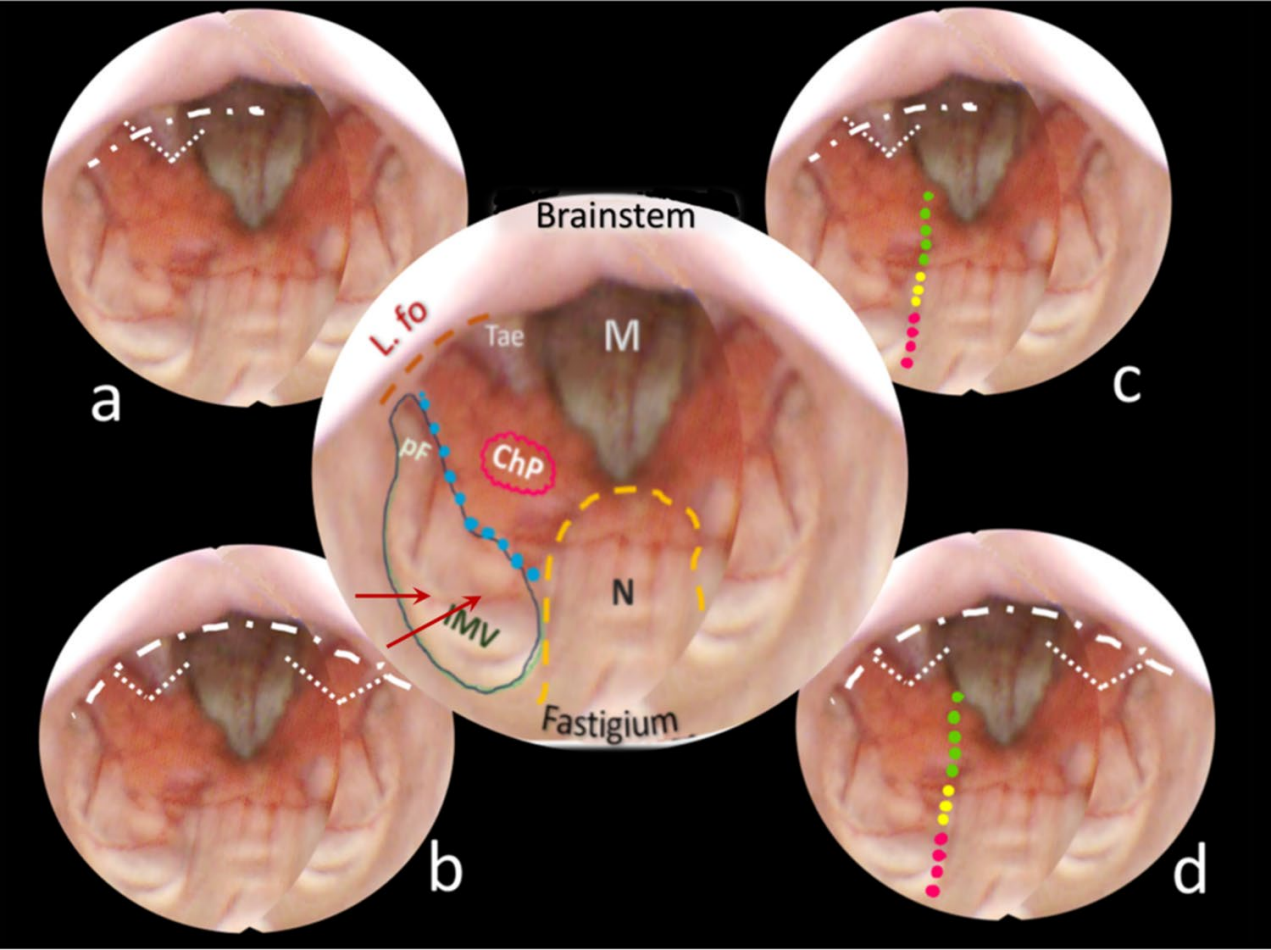


**Fig. 2**


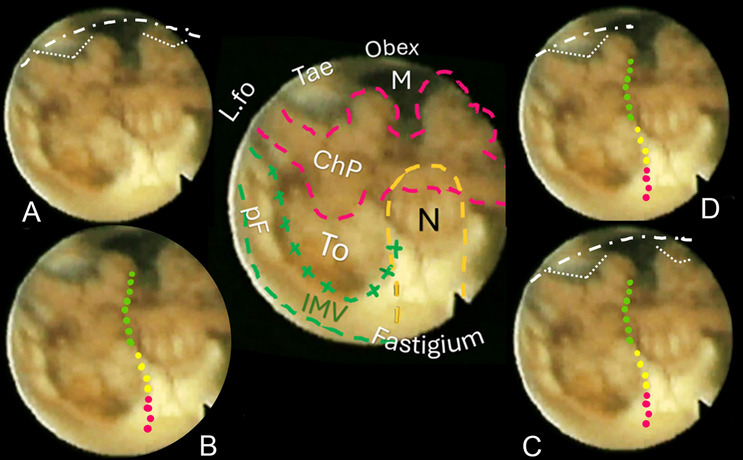


**Fig. 4** Central sketch: anatomical morphology of the roof of the fourth ventricle. The nodulus (N, orange dashed line) lies caudal to the Fastigium, as well as the inferior medullary velum (IMV, outlined in green loosely dashed line), with the pedunculus flocculi (pF) towards the foramen of Luschka (L.fo). The transparent sleeve over the tonsil (To) can be considered the cranial part of a stretched tela, and the green crossed lines outline the tela-velar junction. Caudally, the tela is overshadowed by the choroid plexus (Chp) and is anchored to the brainstem by the two resilient teniae (Tae) that flank the foramen of Magendie (M) on either side. Cranially, their horizontal segment delineates the L.fo. **A** “Lateral recess” approach: section of the vertical and horizontal tract of the taeniae (white loosely dotted lines) that allows detaching the roof from the brainstem, connecting the Magendie to the Luschka foramina with a wide opening on the lateral part of the ventricle (densely dashed-dotted line). **B** The effect of bilateral incision of the teniae is more pronounced. **C** "Extended type" approach. **D** “Lateral wall type” approach


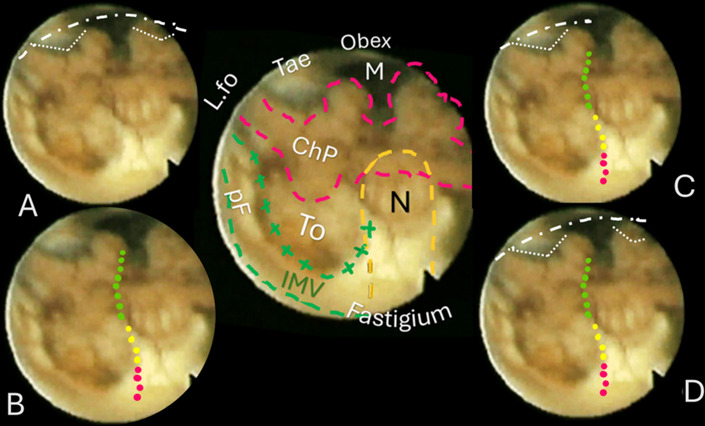


**Fig. 5**

The original article has been corrected.

